# Reperfusion Injury and Infarct Size: Why Translation has Been Difficult, and How We Move Forward

**DOI:** 10.1007/s11886-026-02401-4

**Published:** 2026-08-12

**Authors:** Heerajnarain Bulluck, Derek J. Hausenloy

**Affiliations:** 1 Department of Cardiology, Leeds General Infirmary, Leeds, UK; 2 Leeds Institute of Cardiovascular and Metabolic Medicine, University of Leeds, Leeds, UK; 3 Beacon Hospital, Dublin, Ireland; 4 National Heart Centre Singapore, Singapore, Singapore; 5 Cardiovascular and Metabolic Disorders Program, Duke-NUS Medical School Singapore, 8 College Road, Singapore, 169857 Singapore; 6 Yong Loo Lin School of Medicine, National University Singapore, Singapore, Singapore; 7 The Hatter Cardiovascular Institute, University College London, London, UK

**Keywords:** Myocardial reperfusion injury, Cardioprotection, STEMI, CMR, Intramyocardial haemorrhage, Trial design

## Abstract

**Purpose of Review:**

To evaluate the reasons underlying the translational failure of cardioprotection in reperfused ST-elevation myocardial infarction (STEMI) and propose a framework for designing future clinical cardioprotection trials.

**Recent Findings:**

The 2024 *JACC* Scientific Statement on reperfusion injury reframed the field as a network of interrelated injury pathways but stopped short of providing a clinically actionable framework. Contemporary cardioprotection trials in enriched patient STEMI cohorts have produced largely neutral results: STEMI-DTU, PiCSO-AMI-I, EURO-ICE, and COOL AMI EU failed to demonstrate cardioprotection on cardiovascular magnetic resonance (CMR) infarct size. Although the PITRI trial showed reduced periprocedural platelet reactivity with cangrelor there was no reduction in infarct size or microvascular obstruction (MVO), exemplifying proximal target engagement without affecting imaging surrogates. In contrast, the supersaturated oxygen (SSO₂) programme arc – AMIHOT → AMIHOT-II → IC-HOT → IC-HOT-MICRO – showed progressive patient enrichment yielding a progressive mechanistic signal, particularly in patients with severe coronary microvascular dysfunction. The Collaborative Registry on CMR in STEMI confirmed MVO ≥ 2.6% of left ventricular mass as an independent predictor of heart failure hospitalisation and all-cause death. The EU-CARDIOPROTECTION IMPACT criteria provide a preclinical standard for clinical translation.

**Summary:**

Translational failure of cardioprotection reflects misalignment between heterogeneous biology and trial design, not biological irrelevance. A biological ceiling cannot be excluded but is unlikely to be the dominant barrier. The path forward is to align patient selection, phenotype-specific endpoints, and adaptive trial architecture, and to test mechanical interventions together with pharmacological adjuncts rather than in isolation. When these elements are aligned – as the SSO₂ programme arc suggests – cardioprotection in STEMI may yet deliver clinically meaningful benefit.

## Introduction

Primary percutaneous coronary intervention (PCI) has transformed outcomes in ST-elevation myocardial infarction (STEMI). In-hospital mortality has fallen below 5% in contemporary series, yet the burden of post-myocardial infarction (MI) heart failure (HF) is rising as more patients survive their index event with substantial residual injury [1]. Final infarct size remains a major determinant of left ventricular (LV) remodelling and post-MI HF, and reperfusion itself may contribute up to 50% of final infarct size in experimental and clinical models [2]. There remains, therefore, an unmet need to limit reperfusion injury and reduce final infarct size beyond that achieved by timely revascularisation.

Three decades of cardioprotection trials in reperfused STEMI have been disappointingly neutral. Existing reviews and commentaries have assessed the different strategies – ischaemic conditioning, therapeutic hypothermia, ventricular unloading, supersaturated oxygen, pharmacological adjuncts, and PCI techniques – or mechanisms separately, but rarely integrate the two into a clinically actionable framework. The 2024 *JACC* Scientific Statement on reperfusion injury reframed the field around a network of interrelated injury pathways and acknowledged the translational gap, but stopped short of prescribing how the next clinical cardioprotection trial should be designed [1]. Two recent overviews from Heusch reach a similar conclusion from the bench-side perspective: the field needs new pragmatism rather than new pathways [2, 3]. The clinical reader is therefore left with a description of the problem rather than a route to a more informative clinical cardioprotection trial.

These strategies act on different levers of the same injury network. Ischaemic conditioning, whether applied remotely to a limb or locally to the myocardium, triggers the heart’s endogenous protective signalling through brief, non-injurious cycles of ischaemia and reperfusion. Therapeutic hypothermia slows metabolic demand and mitochondrial calcium handling during the vulnerable early reperfusion window. Ventricular unloading reduces wall stress and myocardial oxygen demand before flow is restored, limiting the metabolic mismatch that reperfusion exposes. Supersaturated oxygen delivers hyperoxaemic blood directly into the infarct-related artery to support microvascular perfusion at the moment of reperfusion. Pharmacological adjuncts target specific nodes in the injury cascade – platelet reactivity, mitochondrial permeability transition pore opening, or inflammation – depending on the agent. PCI technique modifications, including thrombus aspiration and staged or deferred stenting, aim to limit distal embolisation and no-reflow at the point of reperfusion itself. Each therefore addresses a different point in the cascade described in Sect. 2, which is why no single platform has been sufficient on its own.

In this article, we provide an integrated diagnostic assessment of why translation has been challenging, examine four failure modes through a five-point diagnostic, and propose a framework for the next cardioprotection trial in STEMI.

## Defining Reperfusion Injury for the Clinician

Reperfusion injury is best understood as a set of clinically distinguishable phenotypes sharing a common insult – restoration of blood flow to ischaemic myocardium – but differing in mechanisms, time course, and clinical consequence. Lethal reperfusion injury describes cardiomyocyte death triggered by the act of reperfusion itself, distinct from cell death during ischaemia [2]. Reperfusion arrhythmias are typically transient and rarely fatal in the contemporary catheter laboratory. Myocardial stunning is reversible contractile dysfunction in viable myocardium that recovers over days to weeks. Microvascular obstruction (MVO) and the no-reflow phenomenon describe failure to restore tissue-level perfusion despite an open epicardial artery, appearing on cardiovascular magnetic resonance (CMR) as a hypoenhancing core within the infarct zone [4]. Intramyocardial haemorrhage (IMH) is extravasation of red blood cells through a damaged microvasculature, identifiable on T2*-weighted CMR and associated with adverse remodelling and residual myocardial iron deposition [5].

**Fig. 1:**
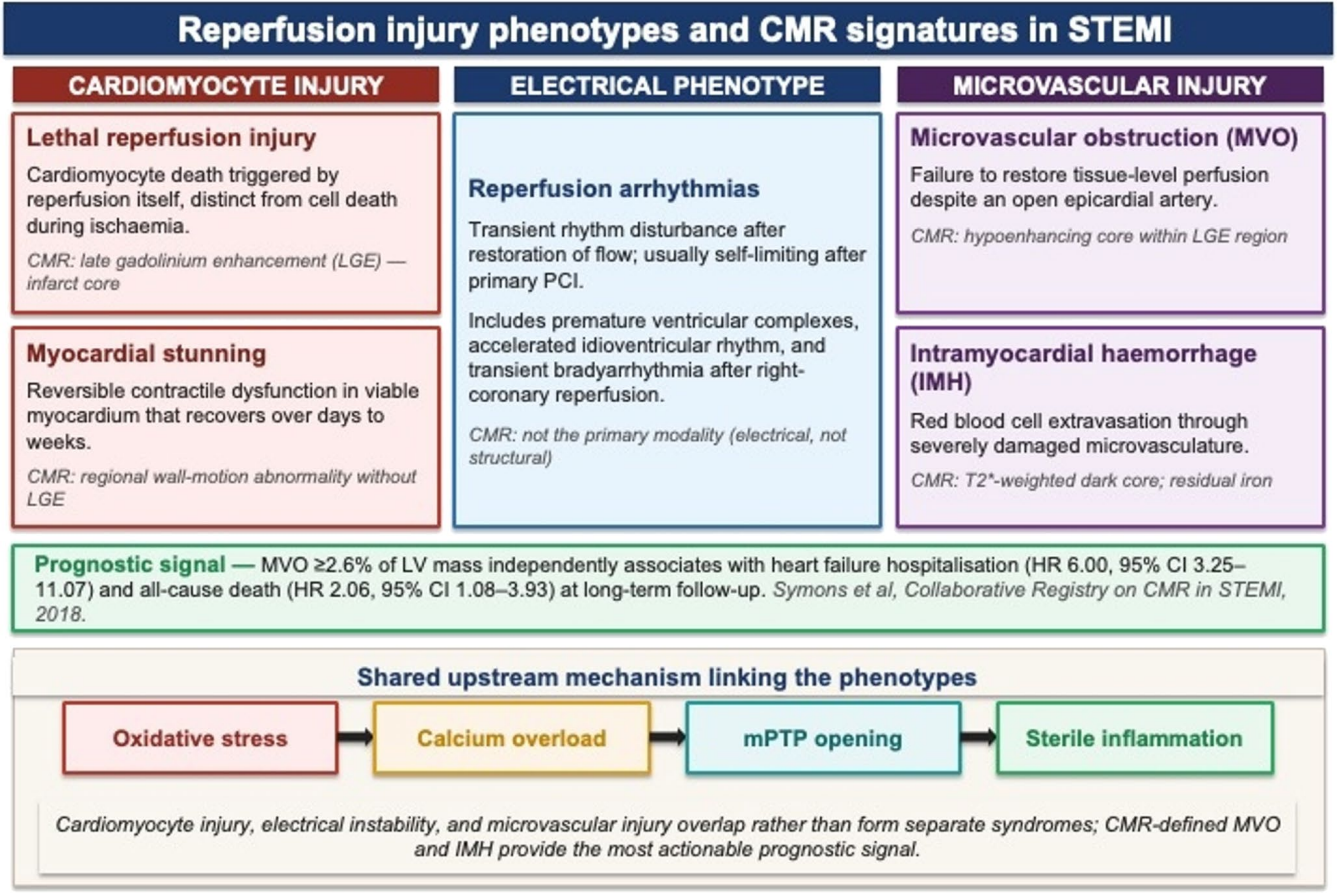
Reperfusion injury phenotypes and CMR signatures in ST-elevation myocardial infarction. Reperfusion injury manifests as a set of clinically distinguishable phenotypes that share a common upstream mechanism. Cardiomyocyte injury includes lethal reperfusion injury – irreversible cell death triggered by reperfusion itself, identified on cardiovascular magnetic resonance (CMR) by late gadolinium enhancement (LGE) [4] and myocardial stunning, reversible contractile dysfunction in viable myocardium with regional wall-motion abnormality but no LGE. The electrical phenotype is characterised by transient reperfusion arrhythmias following restoration of flow. Microvascular injury comprises microvascular obstruction (MVO), seen on CMR as a hypoenhancing core within the LGE region, and intramyocardial haemorrhage (IMH), identifiable on T2*-weighted CMR as a dark core with residual myocardial iron deposition [5]. These phenotypes converge on a shared upstream cascade of oxidative stress, calcium overload, mitochondrial permeability transition pore (mPTP) opening, and sterile inflammation [1, 2]. MVO ≥ 2.6% of left ventricular (LV) mass is independently associated with heart failure hospitalisation and all-cause death [6], representing the most actionable prognostic signal among CMR-derived phenotypes. Abbreviations: CMR, cardiovascular magnetic resonance; IMH, intramyocardial haemorrhage; LGE, late gadolinium enhancement; LV, left ventricular; mPTP, mitochondrial permeability transition pore; MVO, microvascular obstruction; PCI, percutaneous coronary intervention; STEMI, ST-elevation myocardial infarction

Mechanistically, these phenotypes converge on a network of interrelated insults rather than a single pathway: oxidative stress, mitochondrial calcium overload, mitochondrial permeability transition pore (mPTP) opening, sterile inflammation, and progressive microvascular injury [1, 2]. The clinical relevance of these phenotypes is captured by their prognostic implications: in the Collaborative Registry on CMR in STEMI, an MVO extent of ≥ 2.6% of LV mass was independently associated with HF hospitalisation (hazard ratio 6.00, 95% confidence interval [CI] 3.25–11.07) and all-cause death (HR 2.06, 95% CI 1.08–3.93) at long-term follow-up across 810 patients [6]. Figure 1 summarises the phenotypes and their CMR signatures.

## Why Infarct Size Still Matters

Despite advances in revascularisation, secondary prevention, and HF therapeutics, final infarct size remains a strong independent determinant of LV remodelling, HF hospitalisation, and mortality in STEMI survivors [7]. Each 5% absolute increment in infarct size as a percentage of LV mass is associated with adverse changes in LV volumes, ejection fraction, and clinical outcomes. The prognostic signal of MVO sits on top of this: patients with MVO ≥ 2.6% of LV mass carrying an approximately sixfold increased risk of HF hospitalisation independent of infarct size [6]. Reducing final infarct size, and limiting the microvascular component of injury within it, therefore remains a clinically important therapeutic target in STEMI.

## The Paradox of Progress

Modern STEMI care presents a paradox for the cardioprotection field. As primary PCI networks, periprocedural antithrombotic regimens, and rapid reperfusion times have improved, contemporary infarct sizes by CMR have fallen from historical values of 25–30% to 15–20% of LV mass [1, 7]. The absolute room for an adjunctive cardioprotective intervention to demonstrate benefit on a population-level infarct-size endpoint has shrunk accordingly. This reduction has two consequences: it explains, in part, the proliferation of neutral cardioprotection trials over the last fifteen years, and sharpens the case for selective patient enrichment – cardioprotective benefit, where it exists, will be more likely in patients with the greatest residual modifiable injury, not in unselected STEMI populations [2, 3]. This principle underpins the diagnostic that follows.

The question is therefore not whether this therapeutic window has closed, but whether it has simply narrowed to a degree that existing trials are underpowered to resolve. A shrinking absolute effect size reduces a trial’s power to detect benefit; it does not by itself prove that no benefit exists, and a sufficiently large or well-enriched trial could in principle still uncover a real effect that current sample sizes miss. Whether such an effect would matter to patients depends on what it is set against: infarct size itself has no direct meaning to a patient, but Sect. 3 already shows that a 5% absolute increment in infarct size carries measurable prognostic weight for heart failure hospitalisation and death. An intervention too small to shift population-level infarct size detectably could still be worth testing if it shifted these longer-term outcomes, which is why the endpoint hierarchy proposed in Sect. 6.5 anchors on death and heart failure hospitalisation rather than infarct size alone.

The paradox also has a converse. Patients who do not receive the best of contemporary care – because of delayed presentation, prolonged ischaemic time, or a suboptimal angiographic result after primary PCI – retain larger infarct sizes and greater residual modifiable injury than the enriched trial population described in Sect. 6.1. Whether cardioprotective interventions confer differential, and potentially greater, benefit in this less-optimally-treated group is now being tested directly: the RIC-AFRICA trial is evaluating remote ischaemic conditioning in 1200 STEMI patients across sub-Saharan Africa who, by the nature of the healthcare setting, lack access to timely primary PCI [8], reframing benefit in patient-centred rather than population-level infarct-size terms.

## Why Translation has Been Difficult

The Platelet Inhibition To Target Reperfusion Injury (PITRI) trial is one of the clearest contemporary illustrations of this diagnostic problem this section sets out to interrogate [9]. PITRI randomised 209 patients with STEMI undergoing primary PCI to cangrelor or placebo, with CMR infarct size at 4–7 days as the primary endpoint. Cangrelor reduced platelet reactivity approximately twofold during PCI, yet the primary endpoint was unchanged (median infarct size 14.9% [IQR 7.3–22.6] vs. 16.3% [IQR 9.9–24.4] of LV mass, *P* = 0.40) and MVO prevalence was similar (48% vs. 47%, *P* = 0.99) [9]. Proximal target engagement was therefore unequivocal, yet the distal clinical imaging infarct size surrogate endpoint was not reduced. PITRI poses, the question that frames the four failure modes considered below: when an intervention engages its proximal mechanism but did not alter the imaging surrogate, where in the chain of trial design did translation actually fail?

Table 1 summarises these trials, organised by opening case and failure mode.

**Table 1:** Major contemporary cardioprotection trials in reperfused STEMI

Trial (year) [Ref]	Intervention (population)	*n*	Primary endpoint result	Mechanism / reason
Opening case				
PITRI (2024) [9]	IV cangrelor at onset of reperfusion (first STEMI; ticagrelor pre-treated)	209	CMR IS at 4–7 d neutral: median 14.9% (7.3–22.6) vs. 16.3% (9.9–24.4) of LV mass, *P* = 0.40; MVO 48% vs. 47%, *P* = 0.99	Proximal–distal mismatch: target engaged (~ 2-fold reduction in platelet reactivity) without movement in the downstream imaging surrogate
Remote ischaemic conditioning				
RIC-STEMI (2018) [10]	Upper-limb cuff occlusion–reperfusion before primary PCI (single-centre)	258	HR 0.35 (95% CI 0.15–0.78) for cardiac death or HF hospitalisation at median 2.1 years	Regression to the mean: single-centre signal not sustained at scale
CONDI-2/ERIC-PPCI (2019) [11]	Upper-limb cuff before primary PCI (multicentre)	5,401	Cardiac death or HF hospitalisation at 12 mo 8.6% vs. 9.3%; HR 1.09 (0.90–1.32, *P* = 0.38)	Triad misalignment: unenriched STEMI population, clinical-event endpoint constrained by low contemporary event rate, and statistical assumptions anchored to inflated early effect sizes
CONDI-2/ERIC-PPCI CMR substudy (2021) [12]	CMR substudy of CONDI-2/ERIC-PPCI	(subset)	Neutral on 6-mo CMR IS, MVO, and LVEF	Confirmation of neutrality at the imaging endpoint level
Therapeutic hypothermia				
COOL AMI EU (2021) [13]	Systemic endovascular cooling before primary PCI (anterior STEMI)	111	CMR IS 21.3 ± 12.2% vs. 20.0 ± 12.7%, *P* = 0.54; symptom-to-balloon prolonged 44 min (*P* < 0.001); stopped early for futility	Workflow harm: systemic cooling delayed reperfusion sufficient to negate benefit
EURO-ICE (2024) [14]	Selective intracoronary hypothermia at primary PCI (anterior STEMI)	200	3-mo CMR IS 23.1 ± 12.5% vs. 21.6 ± 12.2%, *P* = 0.43; LVEF 49.1 ± 10.2% vs. 50.1 ± 10.4%, *P* = 0.53	Sub-threshold effect: time penalty removed by intracoronary delivery, yet absolute effect remains below the detectable threshold against contemporary background therapy
Mechanical reperfusion modulation				
STEMI-DTU (2026) [15]	LV unloading with Impella CP for 30 min before reperfusion (anterior STEMI without shock)	527	30-d CMR IS mean diff − 1.1% (95% CI −4.2 to 2.0, *P* = 0.50)	Failure of scope: mechanical augmentation alone (arterial side) insufficient to alter the population-level infarct-size distribution
PiCSO-AMI-I (2024) [16]	Pressure-controlled intermittent coronary sinus occlusion alongside primary PCI (anterior STEMI, TIMI 0–1)	145	5-d CMR IS 27.2 ± 12.4% vs. 28.3 ± 11.45%, *P* = 0.59; MVO 67.2% vs. 64.6%, *P* = 0.85; trial prematurely discontinued	Failure of scope: mechanical augmentation alone (venous side) similarly insufficient – not a failure of patient selection or endpoint
Supersaturated oxygen therapy (alignment exemplar – progressive enrichment yields signal)				
AMIHOT (2007) [17]	Intracoronary SSO₂ within 24 h of reperfusion (broad STEMI; anterior or large inferior)	269	Neutral overall; post-hoc benefit confined to anterior STEMI within 6 h	Phenotype dilution: broad enrolment dilutes signal; first hint that efficacy is contingent on patient phenotype and timing
AMIHOT-II (2009) [18]	Intracoronary SSO₂ in anterior STEMI within 6 h (Bayesian RCT, enriched)	301	Median IS 20.0% vs. 26.5% LV mass; posterior probability of superiority 95.1%; non-inferior 30-d MACE	Successful enrichment: controlled positive infarct-size trial demonstrating that progressive enrichment yields a detectable mechanistic signal
IC-HOT (2019, 2021) [19,20]	Optimised SSO₂ via 5 F catheter in left main (anterior STEMI < 6 h, single arm)	100	30-d NACE 7.1% vs. OPG 10.7% (safety primary met); matched-CMR not superior; 1-yr propensity-matched composite (death, new HF, HF hosp) 0.0% vs. 12.3%, *P* = 0.001	Transitional study: optimised delivery confirmed feasible and safe; matched-CMR endpoint less sensitive than enrichment-by-design analyses
IC-HOT-MICRO (2026) [21]	SSO₂ with angio-IMR substudy (anterior STEMI)	50	Median angio-IMR reduced from 44.5 to 23, *P* = 0.003; benefit concentrated in baseline angio-IMR > 40	Mechanism confirmation: SSO₂ acts through reversal of severe coronary microvascular dysfunction, supporting microvascular phenotyping for patient selection

Contemporary cardioprotection trials in reperfused STEMI organised by failure mode, with the contemporary opening case (PITRI), four failure modes (RIC, hypothermia, mechanical reperfusion modulation, SSO₂), and the SSO₂ programme arc as the worked positive trajectory

Key for Table 1: *angio-IMR* angiography-derived index of microcirculatory resistance, *CI* confidence interval, *CMR* cardiovascular magnetic resonance, *HF* heart failure, *HR* hazard ratio, *IS* infarct size, *IV* intravenous, *LV* left ventricular, *LVEF* left ventricular ejection fraction, *MACE* major adverse cardiac events, *MVO* microvascular obstruction, *NACE* net adverse clinical events, *OPG* objective performance goal, *PCI* percutaneous coronary intervention, *RIC* remote ischaemic conditioning, *SSO₂ *supersaturated oxygen, *STEMI* ST-elevation myocardial infarction, *TIMI* Thrombolysis in Myocardial Infarction

This section applies a five-point diagnostic to each failure mode:

Was the right patient population enriched for modifiable injury?

Did the primary endpoint capture the biology the intervention was meant to target?

Was the protocol – timing, dose, route, and duration – aligned to the biology of reperfusion injury?

Were the statistical assumptions anchored to contemporary STEMI infarct-size data, rather than historical ones?

What does any surviving signal reveal about the biology the trial did not target?

### Remote Ischaemic Conditioning: Proof-of-Concept-to-Phase-III Failure

Remote ischaemic conditioning (RIC) provides a clear example of regression to the mean in cardioprotection. The single-centre RIC-STEMI trial randomised 258 patients to upper-limb cuff occlusion–reperfusion before primary PCI and reported a hazard ratio of 0.35 (95% CI 0.15–0.78) for cardiac death or HF hospitalisation at a median of 2.1 years [10]. CONDI-2/ERIC-PPCI then enrolled 5,401 patients to a similar protocol and was neutral at 12 months (cardiac death or HF hospitalisation 8.6% control vs. 9.3% RIC; HR 1.09, 95% CI 0.90–1.32, *P* = 0.38) [11]. The pre-specified CMR substudy was similarly neutral on 6-month infarct size, MVO, and left ventricular ejection fraction (LVEF) [12]. Unlike PITRI, where proximal target engagement was unequivocal, RIC exposes a different failure mode – not mechanism but alignment: an unenriched STEMI population, a clinically-event endpoint constrained by a low contemporary event rate, statistical assumptions anchored to inflated early effect sizes, and uncertain protocol sensitivity to timing and collateral flow. Where signal persists – in patients with longer ischaemic time or anterior infarction – it is insufficient to support a confirmatory phase-III trial in an unselected population, but it remains the most plausible biological residue.

### Therapeutic Hypothermia: Workflow Vulnerability

Therapeutic hypothermia illustrates how protocol execution can negate a biologically plausible intervention. The COOL AMI EU trial randomised 111 anterior STEMI patients to systemic endovascular cooling versus standard care before primary PCI, prolonging symptom-to-balloon time by 44 min (232 ± 63 vs. 188 ± 64 min, *P* < 0.001) and leading to early termination for futility (CMR infarct size 21.3 ± 12.2% vs. 20.0 ± 12.7%, *P* = 0.54) [13]. EURO-ICE then tested selective intracoronary hypothermia at the time of PCI in 200 patients with anterior STEMI, removing the systemic-cooling time penalty, yet remaining neutral on 3-month CMR infarct size (23.1 ± 12.5% vs. 21.6 ± 12.2%, *P* = 0.43) and on LVEF (49.1 ± 10.2% vs. 50.1 ± 10.4%, *P* = 0.53) [14]. The dominant failure axis here was delivery rather than mechanism. Even with the systemic-cooling workflow penalty removed, the absolute incremental effect of intracoronary hypothermia on CMR infarct size was likely below the detectable threshold against contemporary background therapy. Any meaningful effect will require faster onset and earlier application within the ischaemic window than current intracoronary devices and STEMI pathways permit.

### Mechanical Reperfusion Modulation: Well-Designed and Still Neutral

Mechanical reperfusion modulation marks where the diagnostic reaches its limit: two recent trials – one of LV unloading, and one of pressure-controlled coronary sinus occlusion – were neutral despite being well-designed. STEMI-DTU randomised 527 patients to LV unloading with Impella CP for 30 min before reperfusion versus immediate reperfusion, and was neutral on 30-day CMR infarct size (mean difference − 1.1%, 95% CI −4.2 to 2.0, *P* = 0.50) [15]. PiCSO-AMI-I tested pressure-controlled intermittent coronary sinus occlusion alongside primary PCI in 145 patients with anterior STEMI and Thrombolysis in Myocardial Infarction (TIMI) flow grade 0 or 1, and was similarly neutral on 5-day CMR infarct size (27.2 ± 12.4% vs. 28.3 ± 11.45%, *P* = 0.59) and MVO (67.2% vs. 64.6%, *P* = 0.85) before being prematurely discontinued by the sponsor [16]. This holds across both arterial unloading and venous augmentation strategies. Here the failure is one of scope rather than mechanism. When patient selection, endpoint, protocol, and statistical assumptions are each broadly correct, what remains is the proposition that this mechanical augmentation alone did not meaningfully alter contemporary infarct size in reperfused STEMI. A residual benefit, if it exists, will require combining a mechanical intervention with a pharmacological adjunct, on the principle that the platform creates the therapeutic window and the adjunct exploits it.

### Supersaturated Oxygen Therapy: From Neutral Trials to Phenotype-specific Signal

Supersaturated oxygen (SSO₂) therapy delivers hyperoxaemic blood into the infarct-related artery after PCI, with the aim of improving microvascular perfusion, reducing endothelial swelling, and limiting infarct size. AMIHOT enrolled a broad population reperfused within 24 h and was neutral overall, with a pre-specified post hoc signal of benefit confined to anterior STEMI reperfused within 6 h [17]. AMIHOT-II tested this enriched population in a Bayesian randomised controlled trial and demonstrated a significant reduction in infarct size (median 20.0% vs. 26.5% LV mass; probability of superiority 95.1%), with non-inferior 30-day major adverse cardiovascular events [18]. IC-HOT introduced an optimised left-main delivery platform and met its safety primary endpoint (30-day net adverse clinical events 7.1% vs. an objective performance goal of 10.7%), although the matched-CMR comparison did not show infarct-size superiority; a propensity-matched 1-year analysis reported a striking reduction in the composite of all-cause death, new-onset heart failure, or HF hospitalisation (0.0% vs. 12.3%, *P* = 0.001) [19, 20]. IC-HOT should be interpreted not as a negative trial but as a transitional study within the SSO₂ development pathway, confirming feasibility while preserving the earlier infarct-size signal. IC-HOT-MICRO then provided mechanism: SSO₂ reduced the angiography-derived index of microcirculatory resistance (median angio-IMR 44.5 to 23, *P* = 0.003), with benefit greatest in patients with elevated baseline angio-IMR above 40 [21]. A 2024 multi-cohort analysis independently associated SSO₂ with reduced MVO extent (coefficient − 1.35, 95% CI −2.58 to −0.11, *P* = 0.03) [22]. The pattern across the SSO₂ programme is alignment rather than failure: patient selection, endpoint, and protocol converge on a biologically coherent effect. This is what a successful translational signature looks like. The remaining methodological gap is a contemporary randomised trial with a concurrent control arm, powered for hard outcomes in a microvascular-enriched patient cohort; AMIHOT III (a multicentre randomised post-approval trial of SSO₂ versus standard care in 434 patients with left anterior descending (LAD) STEMI within 6 h, evaluating infarct size, microvascular obstruction, heart failure readmission, and quality-of-life outcomes) and REAL SSO₂ (a post-market registry-linked observational comparison of SSO₂ to primary PCI alone in routine anterior STEMI care, evaluating clinical utility and economic value, leveraging the ACC CathPCI registry) have been designed to address this.

### Biological Ceiling Effects: Is Cardioprotection Already “Spent”?

An alternative, and less frequently articulated, explanation is that the capacity for cardioprotection in humans may be intrinsically limited. Unlike preclinical models, patients presenting with STEMI have been exposed to lifelong physiological and pathological stressors, including ageing and other co-morbidities, intermittent ischaemia, and pharmacological therapies, which may induce or affect endogenous conditioning pathways. This raises the possibility that the myocardium at the time of infarction is already partially preconditioned, thereby reducing the incremental benefit achievable with exogenous cardioprotective interventions. In this framework, the repeated failure of clinical trials would reflect a biological ceiling effect rather than deficiencies in trial design alone.

However, this explanation is insufficient to account for the totality of the evidence. First, substantial inter-individual variability in infarct size, microvascular obstruction, and intramyocardial haemorrhage persists despite contemporary care, indicating that modifiable injury remains. Second, several interventions have demonstrated clear biological effects on proximal targets without translating into reductions in infarct size, suggesting that the issue lies not only in biological ceiling but in the alignment between mechanism, measurement, and trial design. Third, signals of benefit observed in enriched populations indicate that cardioprotection may still be achievable when appropriately targeted.

The central question is therefore not whether cardioprotection is universally achievable, but whether it can be demonstrated in the right patients, using the right endpoints, and with protocols matched to the biology of reperfusion injury.

## How We Move Forward: The Triad

### Right Patient – Enrichment

The right patient is the one with the greatest residual modifiable injury at the moment of trial enrolment. In contemporary STEMI, this means anterior infarction, longer ischaemic time (typically symptom-to-balloon ≥ 2 h), pre-PCI TIMI flow grade 0 or 1, and large area-at-risk (AAR). CMR-defined IMH and substantial MVO mark severe microvascular injury and identify the subgroup with the greatest potential for benefit from adjunctive cardioprotection [4–6]. Operationalising this enrichment in real time in the catheter laboratory is increasingly feasible: rapid bedside microvascular indices, including the coronary angiography-derived index of microcirculatory resistance (caIMR) [23], microvascular resistance reserve (MRR) [24], and the coronary Flow Index (coFI) [25], provide estimates of microvascular function within minutes of reperfusion. caIMR and MRR have been validated against the thermodilution-derived index of microcirculatory resistance (IMR) [26]; coFI remains a research tool not yet validated for routine clinical use.

### Right Endpoint – Phenotype-Specific

The right endpoint is one that captures the specific biology the intervention is designed to target, rather than a population-averaged surrogate. Crude infarct size by late-gadolinium enhancement and the myocardial salvage index (MSI) have been the cardioprotection field’s default endpoints for two decades, yet face two conceptual problems. First, myocardial salvage on CMR depends on measuring the extent of myocardial oedema on T2-weighted CMR as a surrogate for the AAR but this parameter may itself be affected by cardioprotective interventions – the AAR-contamination problem [27]. Second, neither captures the microvascular injury phenotype that MVO and IMH represent, despite the strong independent prognostic signal of MVO ≥ 2.6% of LV mass for HF hospitalisation and death [6]. Microvascular injury extends this signal beyond infarct size, acting as an independent determinant of adverse remodelling and heart failure [28]. Phenotype-specific endpoints are therefore preferable to crude MI size [7]: a composite of MVO and IMH on CMR at 3–5 days targets microvascular injury directly; global longitudinal strain (GLS) at 3 months captures sub-clinical contractile dysfunction; and N-terminal pro-B-type natriuretic peptide (NT-proBNP) trajectory at 6 months tracks the haemodynamic consequence of residual injury.

### Right Protocol – Trial Architecture

The right protocol begins before any patient is randomised. A clinical trial of cardioprotection in STEMI should rest on preclinical evidence that meets the IMproving Preclinical Assessment of Cardioprotective Therapies (IMPACT) criteria of the EU-CARDIOPROTECTION COST Action, including independent multicentre replication, comorbidity models (aged, diabetic, hypertensive), and intention-to-treat analysis at the bench [29]. Within the trial itself, three protocol features matter most. Timing of the intervention should be specified relative to reperfusion, with target-engagement substudies to confirm that the proposed mechanism is actually engaged at the proposed dose; benefit appears strongest when delivered early, before microvascular injury is established [30]. Stratification at randomisation by ischaemic time and culprit-territory size balances prognostic injury exposure across arms. Trial architecture should be adaptive, Bayesian, and registry-embedded – using existing platforms such as SWEDEHEART to evolve with the evidence accumulating within the trial.

No adjunctive cardioprotective strategy targeting reperfusion injury – including remote ischaemic conditioning, therapeutic hypothermia, supersaturated oxygen therapy, ventricular unloading prior to reperfusion in stable STEMI, or pressure-controlled intermittent coronary sinus occlusion – has received a class of recommendation in the 2025 American College of Cardiology / American Heart Association (ACC/AHA) acute coronary syndrome guidelines [31]. The only device-related recommendation in this space, for the percutaneous microaxial intravascular flow pump, is Class 2a (Level of Evidence B-R) and restricted to selected patients with STEMI and refractory cardiogenic shock rather than stable reperfused STEMI.

### Combination Thinking

Mechanical augmentation alone has not meaningfully altered infarct size at the population level in contemporary trials. The natural next step is to combine a mechanical platform with a pharmacological adjunct, on the principle that the platform creates the therapeutic window and the adjunct exploits it. PiCSO and SSO₂ create complementary microvascular conditions at reperfusion – PiCSO uses controlled coronary sinus occlusion to redistribute microvascular flow toward the infarct zone; SSO₂ delivers hyperoxaemic blood directly into the infarct-related artery – and either could serve as the platform in such a trial [16–22]. The adjunct layer would target a distinct, time-locked component of reperfusion injury: an anti-inflammatory agent for the inflammatory phase; a mitochondrial-targeted agent for early mPTP opening; and a thrombosis-targeted adjunct for the no-reflow contribution to MVO. This reflects pericyte-mediated capillary constriction, providing a targetable downstream mechanism for combination strategies [28]. Combination trials of this type have not yet been tested in STEMI; designing them is the next logical step from the diagnostic.

### The Next Cardioprotection Trial in STEMI: A Proposed Design Template

The diagnostic and the triad translate into an eight-point template for designing a future cardioprotection trial in reperfused STEMI. Each point is intended to be testable, prescriptive, and applicable to a single platform + adjunct trial.

#### **Preclinical IMPACT gate**

Clinical trials should proceed only with preclinical evidence that meets the IMPACT criteria of the EU-CARDIOPROTECTION COST Action [29] – including independent multicentre replication and validation in comorbidity models (aged, diabetic, hypertensive).

#### Patient enrichment at randomisation

Anterior STEMI, symptom-to-balloon time of 2–6 h, pre-PCI TIMI flow grade 0 or 1, no cardiogenic shock. Where feasible, supplement with rapid pre-PCI biomarker or angiography-derived microvascular triage (caIMR, MRR).

#### Intervention timing

Pre- or peri-reperfusion delivery aligned to the proposed mechanism. A target-engagement substudy should confirm that the proposed mechanism is actually engaged at the proposed dose; without this, a neutral outcome cannot be interpreted with confidence.

#### Endpoint hierarchy

Primary: composite of MVO and IMH assessed by CMR at 3–5 days. Key secondary: infarct size at 6 months, global longitudinal strain at 3 months, NT-proBNP trajectory at 6 months. Expansion phase: death and heart failure hospitalisation at 1 year [4–7].

#### **Adaptive**,** registry-embedded architecture**

Bayesian design with a pre-specified futility look at *n* = 200 on the primary endpoint, expansion to a registry-embedded randomised trial (using SWEDEHEART or equivalent) for the clinical-outcomes phase, with the prior updated across phases.

#### Contemporary statistical assumptions

Sample-size calculations anchored to a contemporary STEMI infarct-size distribution of 15–20% LV mass, not historical 25–30%. Power should be re-derived for the smaller absolute room for benefit, with effect-size assumptions stratified by enrichment criteria.

#### Combination mechanical + pharmacological

A mechanical platform (PiCSO or SSO₂) paired with a pharmacological adjunct targeting a distinct, time-locked component of reperfusion injury, tested together rather than sequentially. The platform creates the therapeutic window and the adjunct exploits it.

#### Reporting standards

IMPACT criteria for the bridging preclinical study; Consolidated Standards of Reporting Trials (CONSORT) for the trial; full pre-registration of the analytical plan; and mandatory reporting of neutral findings.

**Fig. 2:**
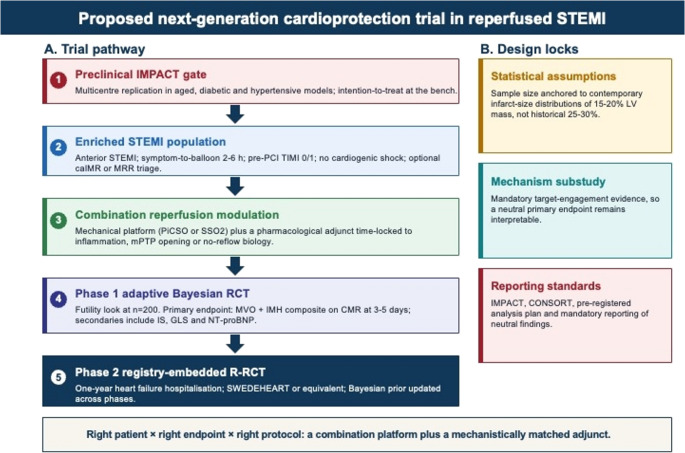
Proposed next-generation cardioprotection trial in reperfused ST-elevation myocardial infarction. The triad framework (right patient × right endpoint × right protocol) is operationalised through a five-stage trial pathway (left) anchored by three transversal design locks (right). The pathway begins with a preclinical IMPACT gate requiring multicentre replication in comorbidity models [29], proceeds through enrichment at randomisation (anterior STEMI, symptom-to-balloon 2–6 h, pre-PCI Thrombolysis in Myocardial Infarction (TIMI) flow grade 0/1, no cardiogenic shock; supplemented by rapid pre-PCI angiography-derived microvascular triage where feasible), and tests combination reperfusion modulation pairing a mechanical platform – pressure-controlled intermittent coronary sinus occlusion (PiCSO) or supersaturated oxygen (SSO2) – with a pharmacological adjunct time-locked to inflammation, mitochondrial permeability transition pore (mPTP) opening, or no-reflow biology. A Phase 1 adaptive Bayesian randomised controlled trial (RCT) with futility look at *n* = 200 takes the composite of microvascular obstruction (MVO) and intramyocardial haemorrhage (IMH) on cardiovascular magnetic resonance (CMR) at 3–5 days as the primary endpoint, with infarct size, global longitudinal strain (GLS), and N-terminal pro-B-type natriuretic peptide (NT-proBNP) trajectory as key secondaries. A Phase 2 registry-embedded RCT (R-RCT) takes one-year heart failure hospitalisation as the clinical-outcomes endpoint, with the Bayesian prior updated across phases. The three transversal locks specify contemporary statistical assumptions (sample size anchored to a contemporary infarct-size distribution of 15–20% of left ventricular (LV) mass, not historical 25–30%), a mandatory mechanism substudy providing target-engagement evidence so that a neutral primary endpoint remains interpretable, and IMPACT and Consolidated Standards of Reporting Trials (CONSORT) reporting standards including pre-registration of the analytical plan and mandatory reporting of neutral findings. Abbreviations: caIMR, coronary angiography-derived index of microcirculatory resistance; CMR, cardiovascular magnetic resonance; CONSORT, Consolidated Standards of Reporting Trials; GLS, global longitudinal strain; IMH, intramyocardial haemorrhage; IMPACT, IMproving Preclinical Assessment of Cardioprotective Therapies; LV, left ventricular; mPTP, mitochondrial permeability transition pore; MRR, microvascular resistance reserve; MVO, microvascular obstruction; NT-proBNP, N-terminal pro-B-type natriuretic peptide; PCI, percutaneous coronary intervention; PiCSO, pressure-controlled intermittent coronary sinus occlusion; R-RCT, registry-randomised controlled trial; RCT, randomised controlled trial; SSO2, supersaturated oxygen; STEMI, ST-elevation myocardial infarction; TIMI, Thrombolysis in Myocardial Infarction

Figure 2 depicts this design as a single trial schematic, and Table 2 contrasts historical with proposed trial-design features.

**Table 2:** Historical vs. proposed trial-design features for cardioprotection in reperfused STEMI

Design feature	Historical trial design	Proposed framework
1. Preclinical evidence gate	Single-centre preclinical efficacy in young, healthy animals; no formal multicentre replication; comorbidity models often absent	IMPACT-validated multicentre preclinical replication with comorbidity models (aged, diabetic, hypertensive); intention-to-treat at the bench [29]
2. Patient selection	Broad STEMI populations (often unselected anterior or inferior); selection on time-to-balloon alone or not at all	Anterior STEMI; symptom-to-balloon time of 2–6 h; pre-PCI TIMI flow grade 0 or 1; no cardiogenic shock; supplemented where feasible by rapid pre-PCI biomarker or angiography-derived microvascular triage (caIMR, MRR) [4–6]
3. Intervention timing	Variable (peri-procedural to post-PCI); rarely with target-engagement substudy; benefit when present often confined to early-presenting subgroups [30]	Pre- or peri-reperfusion delivery aligned to the proposed mechanism; mandatory target-engagement substudy; early administration prioritised before microvascular injury is established [30]
4. Primary endpoint	Crude infarct size by late-gadolinium enhancement at 3–6 months, or myocardial salvage index using T2-weighted area at risk; clinical-event composites in unselected populations	Phenotype-specific composite of MVO and IMH on CMR at 3–5 days, capturing the microvascular-injury phenotype that drives adverse remodelling and HF [6, 28]
5. Secondary endpoints / hierarchy	Single primary endpoint; secondary endpoints often under-powered or post-hoc	Pre-specified hierarchy: infarct size at 6 months, GLS at 3 months, NT-proBNP trajectory at 6 months; expansion phase: HF hospitalisation at 1 year [4–7]
6. Trial architecture	Fixed-sample parallel-group RCT with conventional frequentist statistics	Adaptive Bayesian design with pre-specified futility look at *n* = 200 on the primary endpoint; expansion to a registry-embedded randomised trial (SWEDEHEART or equivalent) for the clinical-outcomes phase; prior updated across phases
7. Statistical assumptions	Sample-size assumptions anchored to historical infarct-size distributions of 25–30% LV mass	Sample-size calculations anchored to contemporary infarct-size distributions of 15–20% LV mass; effect-size assumptions stratified by enrichment criteria
8. Therapeutic strategy	Single-strategy testing (mechanical or pharmacological) in isolation	Combination platform + adjunct: mechanical platform (PiCSO or SSO₂) paired with a pharmacological adjunct targeting a distinct, time-locked component of reperfusion injury (IL-1β blockade, mitochondrial-targeted agent, thrombosis-targeted adjunct, or pericyte-targeted strategy) [16–22, 24]
9. Reporting standards	Variable adherence to CONSORT; under-reporting of neutral findings (publication bias)	IMPACT for any preclinical bridging study; CONSORT for the trial; full pre-registration of analytic plan; mandatory reporting of neutral findings

Historical and proposed trial-design features organised by the eight prescriptive points of Sect. 6.5. The ‘Historical’ column synthesises design choices common to the trials in Table 1; the ‘Proposed’ column operationalises the triad of right patient × right endpoint × right protocol

Key for Table 2: *caIMR* coronary angiography-derived index of microcirculatory resistance, *CMR* cardiovascular magnetic resonance, *CONSORT* Consolidated Standards of Reporting Trials, *GLS* global longitudinal strain, *HF* heart failure, *IL-1β* interleukin-1 beta, *IMH* intramyocardial haemorrhage, *IMPACT* IMproving Preclinical Assessment of Cardioprotective Therapies, *LV* left ventricular, *MRR* microvascular resistance reserve, *MVO* microvascular obstruction, *NT-proBNP* N-terminal pro-B-type natriuretic peptide, *PiCSO* pressure-controlled intermittent coronary sinus occlusion, *RCT* randomised controlled trial, *SSO₂ *supersaturated oxygen, *STEMI* ST-elevation myocardial infarction, *SWEDEHEART* Swedish Web-system for Enhancement and Development of Evidence-based care in Heart disease Evaluated According to Recommended Therapies, *TIMI* Thrombolysis in Myocardial Infarction

## Conclusions

Mortality from reperfused STEMI has fallen below 5% in contemporary series, yet post-infarction heart failure remains a growing burden. Final infarct size remains a major determinant of LV remodelling and HF, and three decades of cardioprotection trials have largely failed to translate into clinical benefit. Cardioprotection in STEMI therefore remains an important unmet need.

The failure to translate a large number of infarct-limiting therapies into the clinical setting should not be taken as evidence that myocardial reperfusion injury does not exist in humans, nor that biological ceiling effects are the dominant barrier. Rather, it should provide the impetus to align patient selection, endpoint choice, and trial architecture with the biology of reperfusion injury. It should also prompt the testing of mechanical platforms together with pharmacological adjuncts rather than in isolation. When these three are aligned – as the SSO₂ programme arc suggests when applied progressively and tested under these conditions – cardioprotection in STEMI may yet deliver clinically meaningful benefit.

### Key References

- Bulluck H, Chong JH, Bryant J, Chai P, Chawla A, Chua TS, et al. Effect of Cangrelor on Infarct Size in STEMI Treated by PPCI: The PITRI Trial. Circulation. 2024;150(2):91-101.
  - ◦This trial is the opening case for the diagnostic framework proposed in this review, showing that unequivocal proximal target engagement does not guarantee a change in the downstream imaging endpoint.
- Welt FGP, Batchelor W, Spears JR, Penna C, Pagliaro P, Ibanez B, Drakos SG, Dangas G, Kapur NK. Reperfusion Injury in Patients With Acute Myocardial Infarction: JACC Scientific Statement. J Am Coll Cardiol. 2024;83(22):2196-2213.
  - ◦ This scientific statement sets out the contemporary clinical framing of reperfusion injury against which the five-point translational diagnostic in this review is applied.
- Kapur NK, Mangner N, Aghili N, et al. Left Ventricular Unloading in Anterior STEMI Without Shock: The STEMI Door to Unload Randomized Controlled Trial. J Am Coll Cardiol. 2026; Epub ahead of print.
  - ◦This trial illustrates that a well-designed, mechanistically sound intervention can still be neutral, reinforcing this review's central argument that trial design alone does not guarantee translation.

## Data Availability

No datasets were generated or analysed during the current study.
